# SARS-CoV-2 compromises blastocyst quality by modifying the ovarian microenvironment

**DOI:** 10.1038/s41392-025-02156-4

**Published:** 2025-02-18

**Authors:** Chen Geng, Min Zhang, Ning Wang, Mei Li, Linlin Cui

**Affiliations:** 1 The Second Hospital of Shandong University, Institute of Women, Children and Reproductive Health, Shandong University, Jinan, China; 2 State Key Laboratory of Reproductive Medicine and Offspring Health, Center for Reproductive Medicine, Institute of Women, Children and Reproductive Health, Shandong University, Jinan, Shandong China

**Keywords:** Biochemistry, Clinical genetics, Industrial microbiology

**Dear Editor**,

The coronavirus disease 2019 (COVID-19) pandemic, caused by severe acute respiratory syndrome coronavirus 2 (SARS-CoV-2), has raised concerns about its impact on female reproductive functions.^1^ While direct viral damage to ovarian cells is limited,^2,3^ the immune response to COVID-19 may cause organ damage,^4^ and its impact on female reproductive capacity remains unclear.

To investigate changes in the ovarian microenvironment due to COVID-19, we analyzed transcriptomics and proteomics of granulosa cell, as well as metabolomics and lipidomics of follicular fluid (FF), in 9 COVID-19 patients and 17 non-COVID-19 controls. Nine genes and proteins were upregulated in both transcriptome and proteome state, including Myxovirus resistance 1 (MX1), interferon-stimulated gene 15 (ISG15), interferon induced protein with tetratricopeptide repeats 1(IFIT1), IFIT3, interferon alpha inducible protein 27 (IFI27), 2’-5’-Oligoadenylate Synthetase 2 (OAS2), cytidine monophosphate kinase 2 (CMPK2), complement C1q C chain (C1QC) and C1QB (Fig. 1a). Among the nine overlapping differential expression proteins (DEPs), MX1 and ISG15 showed the greatest disparity (Fig. 1a). Kyoto Encyclopedia of Genes and Genomes (KEGG) pathway analysis revealed differentially expressed genes (DEGs) and DEPs enrichment in infection-related pathways, such as Coronavirus disease of COVID-19, Human papillomavirus infection, Hepatitis C, and Influenza A (Fig. 1d). Quantitative real-time polymerase chain reaction and western blot confirmed upregulated MX1 and ISG15 in COVID-19 patients compared to controls (Fig. 1a).

**Fig. 1:**
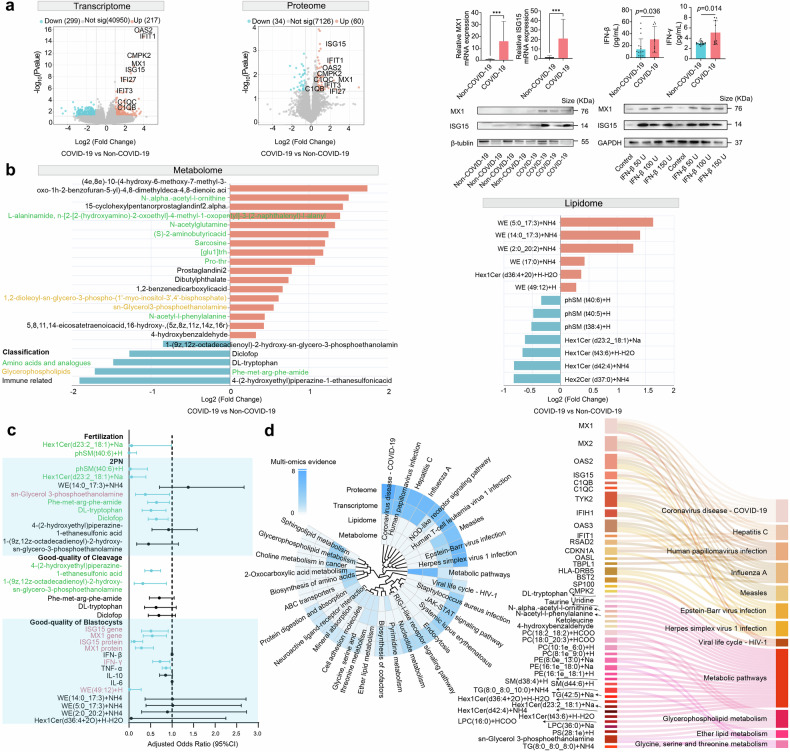
Multi-Omics and immune profiles in the ovarian microenvironment of COVID-19 patients. **a** Volcano plots comparing COVID-19 with non-COVID-19 in transcriptome and proteome, highlighting overexpressed (orange) and underexpressed (blue) genes. qRT-PCR and Western blot of granulosa cells are shown in both COVID-19 and non-COVID-19 groups. IFN-β and IFN-γ levels in follicular fluid. Western bolt of KGN cells incubated with IFN-β. **b** Differential metabolites with higher concentrations in orange, lower in blue. Font color indicates classification. The relative abundance of lipids is determined by the significant lipids with altered abundance. **c** Correlations of genes, proteins, immune factors, metabolites and lipids with oocyte competency. Edge width shows 95% CI of adjusted odds ratio. Blue lines indicate adjusted *p* < 0.05. Green font indicates molecules beneficial for oocyte competency, while red font indicates harmful ones. The generalized estimating equations models are adjusted for female age, BMI, stimulation protocols, gonadotropin dose, and stimulation duration. **d** Multi-omics enriched KEGG pathways; color represents multi-omics evidence; multi-omics molecules associated with KEGG pathways

We evaluated immunological features after SARS-CoV-2 infection and found elevated interferon β (IFN-β) and IFN-γ levels in COVID-19 follicular fluid compared to the controls (Fig. 1a). The addition of IFN-β increased expression levels of MX1 and ISG15 (Fig. 1a). Using the generalized estimating equations (GEE) model after adjusting for potential confounders, higher MX1 and ISG15 expression levels were associated with decreased odds of good-quality blastocysts (Fig. 1c). This implies that the interferon response may affect granulosa cell by regulating MX1 and ISG15.

Key differentially abundant metabolites included immune-related metabolites, glycerophospholipids, and amino acids and analogues. Diclofop, DL-trypotopan, 1-(9z,12z-octadecadienoyl)-2-hydroxy-sn-glycero-3-phosphoethanolamine, 4-(2-hydroxyethyl) piperazine-1-ethanesulfonic acid, and Phe-met-arg-phe-amide were lower in COVID-19 FF compared to controls (Fig. 1b). GEE analysis showed that lower levels of 1-(9z,12z-octadecadienoyl)-2-hydroxy-sn-glycero-3-phosphoethanolamine, and 4-(2-hydroxyethyl) piperazine-1-ethanesulfonic acid were associated with increased odds of good-quality cleavages (Fig. 1c). Similarly, lower level of Diclofop, DL-trypotopan, and Phe-met-arg-phe-amide were linked to increased odds of two-pronuclear zygotes (2PN) (Fig. 1c). In contrast, sn-glycerol-3-phosphoethanolamine was increased in COVID-19 FF and was associated with a lower 2PN rate. These metabolites were mainly enriched in Glycerophospholipid metabolism, 2-Oxocarboxylic acid metabolism, Biosynthesis of amino acids and ATP-binding cassette (ABC) transporters (Fig. 1d).

The lipid profile of the FF was assessed. Wax exters (WE) (49:12) + H was elevated in COVID-19 FF (Fig. 1b) and linked to lower odds of good-quality blastocysts (Fig. 1c). Conversely, Sphingomyelin (phSM) (t40:6) + H and Hexosyl1 ceramide (Hex1Cer) (d23:2_18:1)+Na was lower in FF of COVID-19, associated with higher odds of fertilization and 2PN (Fig. 1c). Differential lipids were enriched in sphingolipid metabolism, glycerophospholipid metabolism, and choline metabolism in cancer (Fig. 1d). SARS-CoV-2 infection caused changes in metabolites and lipids, some of which were detrimental to oocyte competency, while others were beneficial.

An integrated analysis of RNA-seq, protein profiles, lipids and metabolites identified enriched pathways (Fig. 1d). Top co-enriched pathways in RNA-seq and protein profiles included Coronavirus disease -COVID-19, Human papillomavirus infection, Hepatitis C, and Influenza A pathways. The Pyrimidine metabolism pathway was enriched in metabolomics, transcriptomics, and proteomics. Glycerophospholipid metabolism, Glycine, serine and threonine metabolism, and Ether lipid metabolism were shown in lipidomics and metabolomics. Figure 1d also illustrated the involvement of RNA, proteins, lipids, and metabolites in these pathways.

Overall, after SARS-CoV-2 infection, increased IFN-β levels in FF stimulated MX1 and ISG15 expression. The quality of oocytes and their cumulus cells is crucial for embryo potential.^5^ Both ISG15 and MX1, along with elevated levels of sn-glycerol-3-phosphoethanolamine and WE (49:12) + H, negatively impacted blastocyst quality and 2PN rate. Luckily, metabolites and lipids like Diclofop, DL-tryptophan, 1-(9z,12z-octadecadienoyl)-2-hydroxy-sn-glycero-3-phosphoethanolamine, 4-(2-hydroxyethyl) piperazine-1-ethanesulfonic acid, Phe-met-arg-phe-amide, phSM (t40:6) + H, and Hex1Cer(d23:2_18:1)+Na had positive effects to oocyte competency (Fig. 1c). We identified multi-omics changes distinguishing oocyte competence biomarkers post-SARS-CoV-2 infection, aiding diagnostic tool development. Our finding highlights the complex influence on oocyte competency of gene expressions, including ISG15 and MX1, and alterations in metabolites and lipids. These observations, including the need for further animal experiments, warrant additional investigation with a large sample size.

## Supplementary information

SARS-CoV-2 compromises blastocyst quality by modifying the ovarian microenvironment

## Data Availability

Transcriptomic data (GSE282892) are on GEO. Proteomics data (PXD058234) are on ProteomeXchange. Metabolomics (MTBLS11756) and lipidomics (MTBLS11755) data are on MetaboLights.
