# Supplementary material for: SARS-CoV-2 compromises blastocyst quality by modifying the ovarian microenvironment
Source: Signal Transduct Target Ther. 2025 Feb 18;10:68. doi: 10.1038/s41392-025-02156-4 (PMC11836307; doi:10.1038/s41392-025-02156-4)
Supplement: Supplementary file 1 — SARS-CoV-2 compromises blastocyst quality by modifying the ovarian microenvironment [file 41392_2025_2156_MOESM1_ESM.docx]

Supplementary Materials for

**SARS-CoV-2 influences oocyte competency by modifying the ovarian microenvironment**

Chen Geng ^1, 2^†, Min Zhang^1, 2^†, Ning Wang^1, 2^, Mei Li^1, 2^*, Linlin Cui^1, 2^*

* Correspondence to: [fdclear3@126.com](mailto:fdclear3@126.com) or [lee_mei@163.com](mailto:lee_mei@163.com)

**This file includes:**

Materials and Methods

**Materials and Methods**

**Patients:** Nine COVID-19 and 17 non-COVID-19 participants from the Second Hospital of Shandong University underwent IVF or ICSI cycles due to various infertility factors between December 22, 2022 and March 4, 2023. COVID-19 diagnoses were confirmed via nasopharynx swab RT-PCR tests. COVID-19 participants were infected during controlled ovarian stimulation; non-COVID-19 participants had never been infected. Inclusion criteria for non-COVID-19 participants included normal menstrual cycles, endocrine levels, and ovarian/uterine morphology. Exclusions were women over 40, with autoimmune disorders, chromosome abnormalities, adenomyosis, endometriosis, polycystic ovarian syndrome, ovarian surgery, radiotherapy, chemotherapy, or incomplete data. This study was approved by the ethics committee (KYLL-2022-581).

Embryo quality was assessed by two embryologists according to morphological criteria. Fertilization stages include 1pronucleus (PN), 2PN, and 3PN. Mature follicles on the trigger day= No. follicles ≥14 mm present on the day of HCG administration; fertilization rate= (2PN + 3PN)/No. inseminated; good-quality cleavage rate = No. good-quality cleavage/No. inseminated; good-quality blastocyst rate = No. good-quality blastocyst /No. inseminated. Good-quality cleavage stage embryos (day 3) had 7-10 cells and are at the 3' or 4' levels or compact stage. Good-quality blastocysts (days 5-7) had a grade of 4, 5, or 6 and a sub-grade of 'A' or 'B'.

**Human tissues:** Ovarian granulosa cells (GCs) and follicular fluid (FF) were collected from each participant on oocyte retrieval day. Large follicles (≥14 mm) were collected from the FF, which contained mural GCs. The FF was centrifuged (2,000× g for 10 min) and then the supernatants were obtained for untargeted metabolomics and lipidomics. Virological levels of FF were detected by qRT-PCR, and the immunological features, including interleukin (IL)-4, IL-6, IL-10, Tumor necrosis factor (TNF)-α, and Interferon (IFN)-γ and IFN-β, were tested by enzyme-linked immunosorbent assay (ELISA). The precipitates were treated with hyaluronidase (80 IU/ml; Solarbio, 37326–33-3), transferred to lymphocyte separation medium (TBDsciences, LTS1077), centrifuged (1,600× g for 10 min), and the GCs interlayer phase was collected, washed with PBS, and centrifuged again. The GCs were used for transcriptomics, proteomics analysis and qRT-PCR. And another four COVID-19 and four control GCs samples were used for western blots.

**DEG and DEP analysis:** The multi-omics data were conducted by Shanghai Applied Protein Technology Corporation. The quality control samples were created by pooling of aliquots from each individual sample of interest and were measured between repeated measurements of clinical samples. In the transcriptomics and proteomics experiment, DESeq2 (v 1.32.0) R software package was used for analyzing differentially expressed genes (DEGs) and proteins (DEPs). Fold change> 1.5 or <0.667 and *p*-value<0.05 were considered as DEPs and fold change> 2 or <0.5 and *p*-value<0.05 were considered as DEGs. Results were shown using volcano plots generated with Bioconductor in R. The heatmap was created using the ComplexHeatmap (v 2.8.0) package in R.

**Metabolite and** **lipidomic data for analysis and processing:** Metabolomic data (MTBLS11756) and lipidomic data (MTBLS11755) are available on MetaboLights. Specific methods can be found in the database. Data were normalized to total peak intensity and uploaded to MetaboAnalyst (<https://www.metaboanalyst.ca/>) for PCA and OPLS-DA analysis. Model robustness was evaluated using 7-fold cross-validation and response permutation testing. VIP values >1 and *p*-values <0.05 were considered statistically significant. Significance was determined using an unpaired Student’s *t*-test.

**Enrichment analysis:** Gene Ontology (GO), Kyoto Encyclopedia of Genes and Genomes (KEGG) enrichment analysis were performed to find enriched molecular mechanisms and cellular functions of the obtained DEGs. We used clusterProfiler R (v 4.0.5) software package for GO function and KEGG pathway enrichment analysis. GO or KEGG function was significantly enriched when adjusted *p*<0.05.

**RNA extraction and qRT-PCR:** 1 mL of TRIzol Reagent (Thermo Scientific, USA) was added to each tube of ovarian granulosa cells, and RNA was extracted per the TRIzol protocol. RNA purity (A260/A280 ratio) and concentration were measured using a Nanodrop ND-2000 (Thermo Scientific, USA). RNA integrity number (RIN) was assessed with an Agilent Bioanalyzer 4150 (Agilent Technologies, CA, USA). Only samples meeting quality criteria were used for library construction.

Evo M-MLV RT-PCR reverse transcriptase Kit (Accurate Biotechnology, China) was used to reverse transcription of the RNA, and SYBR Green was detected by qRT-PCR with GAPDH as the reference. The forward primer of ISG15 sequence was 5’-GGTGGACAAATGCGACGAAC-3’, and the reverse primer was 5’- TCGAAGGTCAGCCAGAACAG-3’. The forward primer of MX1 sequence was 5’- GGATCGTGACCAGATGCCC-3’, and the reverse primer was 5’- CTGATTCCCATTCCTTCCCCG-3’. The forward primer of GAPDH sequence was 5’-GCACCGTCAAGGCTGAGAAC-3’, and the reverse primer was 5’-TGGTGAAGACGCCAGTGGA-3’. The 2^−△△Ct^ method was used to determine mean relative gene expression.

**Western blots:** Equal amounts of protein were separated on SDS-PAGE gels. The membranes were incubated overnight at 4°C with the following primary antibodies: ISG15 (Abcam, AB285367, 1:1000), MX1 (Abcam, AB207414, 1:2000), β-Tubin (Abmart, M20005, 1: 5000), GAPDH (Proteintech, 60004, 1:5000). Horseradish peroxidase-labelled secondary antibodies (goat anti-rabbit IgG (H+L) (1:5000); goat anti-mouse IgG (H+L) (1:5000), (CST) were used, band signals were detected using chemiluminescent substrate (Thermo Fisher Scientific Inc) and quantified using ImageJ software (National Institutes of Health). β-Tubin and GAPDH bands were used for normalization.

**Statistical analysis:** Normally distributed values are reported as means (standard deviation) and compared using the Student’s *t*-test; skewed data are reported as median (interquartile range) and compared using the Wilcoxon rank-sum test. Categorical variables are reported as frequencies and percentages and compared by means of the Fisher's exact test. Pearson’s correlation analysis was performed to determine the correlation amongst all variables. The generalized estimating equation (GEE) model was used to compare associations between the transcription and protein expression levels of MX1 and ISG15 genes, different metabolites, and the clinical parameters. These parameters include 2PN formation, fertilization, good-quality cleavage-stage embryos, and good-quality blastocysts. The female age, female body mass index, ovarian stimulation protocols, total gonadotropin dose, and duration of ovarian stimulation were added to the adjusted model. SPSS 27.0 software was used for the statistical analyses, with *p*-values less than 0.05 considered significant.
